# High-intensity interval training among middle-aged and older adults for body composition and muscle strength: A systematic review

**DOI:** 10.3389/fpubh.2022.992706

**Published:** 2022-09-29

**Authors:** María Alzar-Teruel, Agustín Aibar-Almazán, Fidel Hita-Contreras, María del Carmen Carcelén-Fraile, Antonio Martínez-Amat, José Daniel Jiménez-García, Raquel Fábrega-Cuadros, Yolanda Castellote-Caballero

**Affiliations:** Department of Health Sciences, Faculty of Health Sciences, University of Jaén, Jaén, Spain

**Keywords:** high-intensity interval training (HIIT), middle-aged, older adults, body composition, muscle strength, systematic review

## Abstract

**Background:**

The aging of population is leading to the investigation of new options to achieve healthy aging. One of these options is high-intensity interval training (HIIT), although its effects on body composition and muscle strength are currently unclear. The objective of this systematic review is to examine the scientific publications on the effects of HIIT on the body composition and muscle strength of middle-aged and older adults.

**Methods:**

The search was carried out in the PubMed, Cochrane Plus, Web of Science, CINAHL and SciELO databases without limitation of publication dates. The literature search, data extraction and systematic review were performed following the PRISMA standards and the risk of bias of the selected studies was assessed using the Cochrane Collaboration Risk-of-Bias.

**Results:**

Initially 520 publications were identified, out of which a total of 8 articles were finally selected to be included in this systematic review. Improvements in body composition were seen in six of the selected items and an increase in muscle strength in seven of the eight. Regarding physical function, improvements were found in both gait speed and balance.

**Conclusions:**

This systematic review found that HIIT is effective in improving body composition and increasing muscle strength. However, when comparing HIIT to moderate-intensity continuous training, it is not clear that HIIT is more beneficial; a firm conclusion cannot be drawn due to the scarcity of published studies, their variety in methodology and the ambiguity of their results, so it is suggested to carry out more research in this area.

## Introduction

Worldwide, the demographic trend toward an aging population is having far-reaching social and financial consequences (1). It is expected that by the year 2050 the aged will have surpassed in number those between the ages of 10 and 24 years old (2). As a matter of fact, in 2018 the percentage of the elderly among the Spanish population was 18.5%, and projections indicate that it could reach 35.5% of the total by 2050 (3). From the point of view of health, the aging process is associated with a variety of complications which include cognitive and functional deterioration, gait alterations, an increased number of falls, greater fragility, as well as the associated increase in disability and dependency (4, 5).

Aging involves an increase in fat deposits between and within muscles. This growth of intramuscular adipose tissue and its lipotoxic effect have been considered to contribute to decreases in muscle quality and strength, given that the infiltration of fat in muscles may alter the orientation of fibers and, as a consequence, the muscle's ability to exert force (6, 7). Additionally, obesity is linked to several non-communicable diseases, among which diabetes, some types of cancer, and cardiovascular diseases stand out (8). This leads to a decrease in life expectancy and higher mortality rates for those affected (9, 10). This situation, however, can be resolved or ameliorated through weight loss and the adoption of healthy lifestyle habits (10).

Sarcopenia is yet another common complication associated with aging. Defined in 2019 by the European Working Group on Sarcopenia in Older People as a decrease in muscle strength as the main diagnostic criterion, sarcopenia is also characterized by a decrease in the quantity or quality of muscle mass. It is considered severe when poor physical performance is added to the criteria mentioned above. Sarcopenia is associated with an increase in the number of falls and fractures, as well as with decreased quality of life, physical disability, and mortality (11).

Physical activity has been shown to provide great benefits for the physical and mental health of older people (12). It has also been proven to increase their quality of life and functional independence, and to decrease their risk of mortality, thus increasing life expectancy (13).

When many individuals consider getting involved in a physical exercise program, lack of time is often cited as a main hurdle. One possible solution to this problem is provided by high-intensity interval training (HIIT), which combines high-intensity intervals with rest or low-intensity periods (14). HIIT allows training to be performed in shorter bouts of time, and is commonly regarded to be more fun and pleasant than moderately intense continuous training. Additionally, HIIT appears to induce more physiological benefits than other traditional kinds of training, and to require shorter training periods (15). Furthermore, HIIT has been reported to be safe and effective for a healthy older population (16).

Despite the health benefits of HIIT and the widespread need to devise and implement active aging plans, to date little research has been done regarding the effects of high-intensity interval training among older and middle-aged people. To the best of our knowledge few studies have focused on measuring body composition and muscle strength, and those that did failed to analyze healthy populations (17). The main goal of this systematic review is to provide an analysis of what data has been published regarding the effects of high-intensity interval training on the body composition and muscle strength of middle-aged and older adults.

## Materials and methods

The bibliographic search, data extraction, and systematic review were carried out in compliance with PRISMA guidelines.

### Eligibility criteria

The inclusion criteria for this systematic review were as follows: Randomized Controlled Trials (RCTs) in which at least one group of the study participated in a HIIT program; that studied the effects of HIIT on obesity and muscle strength; conducted on healthy participants over 55 years old; and published in either English or Spanish. Studies were excluded if: they did not include a control group; or their participants were taking vitamin or protein supplements that might have influenced the results of the study.

### Information sources and search strategy

A systematic literature search was performed in the PubMed, Cochrane Plus, Web of Science, CINAHL, and SciELO databases without any limitation to publication dates. A search was conducted in the title and abstract fields by entering the free terms (“high intensity interval”) AND (“body composition“ OR obes^*^ OR fat OR adipos^*^ OR ”body mass") AND (strength OR “muscle strength” OR “muscle quality”) AND (old OR older OR elder^*^ OR aging OR aging OR aged OR menop^*^ OR postmenop^*^). An iterative process was employed to assure that all relevant articles were selected. The search was conducted from May 2, 2021 to August 16, 2021.

### Study selection and data extraction

The study selection was carried out independently by three of the authors (AAA, FHC, MAT). First, duplicate articles were removed. Then, titles and abstracts were examined to reject the articles that did not meet the eligibility criteria mentioned above. Lastly, full-text articles were screened to confirm that they met the inclusion criteria. Disagreements were solved by discussion until consensus was reached. For each paper, data were extracted concerning authors, year, country, studied population (age, sample size, and group allocation), study design, outcomes, measuring tools used, description of the intervention procedures (type of HIIT and duration), measurement time points, dropout rate by groups, adverse effects, and main findings.

### Outcomes

The primary outcomes of this study were muscle strength and body composition, including body mass index (BMI), body weight, fat mass, or fat-free mass. Secondary outcomes included physical function or physical performance.

### Study quality

The risk of bias of the studies selected was assessed independently by three authors (MCF, AMA, JDJG) using the Cochrane Collaboration Risk-of-Bias tool (18). Any disagreements regarding methodological quality were resolved by discussion until a consensus was reached. The items included in the quality assessment were: selection bias (random sequence generation and allocation concealment), performance bias (blinding of participants and personnel), detection bias (blinding of outcome assessment), attrition bias (incomplete outcome data), reporting bias (selective reporting), and other potential biases. Each item was categorized into one of three levels: low risk (unlikely to alter the results), unclear risk (no specific details or description were reported; raises doubts about the results), or high risk (did not meet the criteria; may alter the results seriously).

## Results

### Included studies

**Figure 1:**
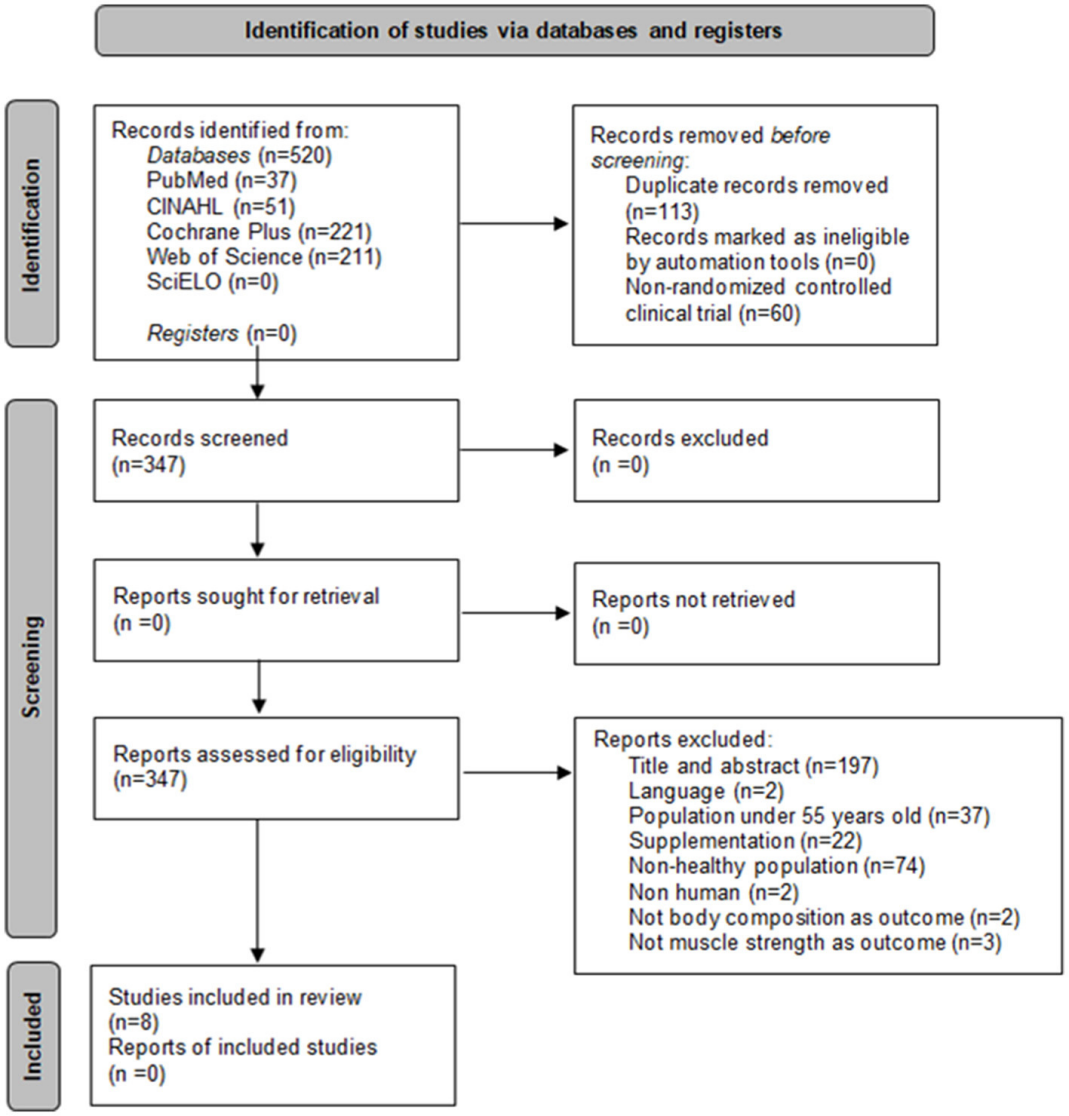
PRISMA flowchart showing the inclusion and exclusion of studies in the systematic review.

In the first search 520 publications were identified, out of which 8 articles were finally selected to be included in this systematic review. Figure 1 shows the study selection flowchart, in accordance with the PRISMA (19) statement.

### Quality of included studies

Table 1 shows the risk-of-bias assessment. All included articles were classified as having a low risk of bias in all items. The eight RCTs included in this systematic review described the exclusions and losses to follow-up (16, 20–26). Only two RCTs did not present exclusions or losses to follow-up.

**Table 1:** Assessment of risk of bias for included studies.

**Articles**	**Random sequence generation (selection bias)**	**Allocation concealment (selection bias)**	**Blinding of participants and personnel (performance bias)**	**Blinding of outcomes assessment (detection bias)**	**Incomplete outcome data (attrition bias)**	**Selective reporting (reporting bias)**	**Other bias**
Taaffe et al. (20)	U	U	H	H	L	L	L
Nemoto et al. (21)	L	H	H	H	U	L	L
Villanueva et al. (22)	U	U	H	U	L	L	L
Sculthorpe et al. (23)	L	U	H	U	L	L	L
Moro et al. (24)	L	L	H	H	U	L	L
García-Pinillos et al. (16)	U	H	H	H	U	L	L
Jiménez-García et al. (25)	L	L	H	L	L	L	L
Ballesta-García et al. (26)	L	L	H	L	L	L	L

L, Low risk; H, High risk; U, Unclear.

### Study and participants characteristics

Table 2 shows the full descriptive details of the RCTs included in this review. Out of the eight articles analyzed, four were two-armed trials (16, 22–24), three were three-armed (21, 25, 26), and the remaining one had four arms (20). Two RCTs were conducted in America (both in California, United States) (20, 22), five in Europe (one in Italy, three in Spain, and one in the United Kingdom) (16, 23–26), and one took place in Asia (Japan) (21).

**Table 2:** Summary of included studies (*n* = 8).

**Study, year, and location**	**Studied population, groups, and study design**	**Outcomes and measuring tools**	**Intervention**	**Measure time points, dropout, and adverse effects**	**Main findings**
Taaffe et al. (20) 1999 California, United States	53 healthy community-dwelling adults (65–79 years) (19 women; 34 men) IG1 (*n* = 14, 68.5 ± 3.6 years; 5 women, 9 men) IG2 (*n* = 14, 69.4 ± 3 years; 4 women, 10 men) IG3 (*n* = 11, 71 ± 4.1 years; 4 women, 10 men) CG (*n* = 14, 68.9 ± 3.6 years; 6 women, 8 men) Design: Randomized controlled trial.	Primary outcome Muscle strength: 1-RM Body Composition: Lean mass, fat mass (DXA) Secondary Outcome Physical Function: Balance (6-meter backward tandem walk) Muscle performance (Chair-rise test)	IG1: 24 weeks. High-intensity resistance training 1 day per week. (Minimum of 30-s rest between sets and at least 2 min between exercises) IG2: 24 weeks. High-intensity resistance training 2 days per week. (Minimum of 30-s rest between sets and at least 2 min between exercises) IG3: 24 weeks. High-intensity resistance training 3 days per week. (Minimum of 30-s rest between sets and at least 2 min between exercises) CG: Inactive	Measurements: At baseline After the intervention Dropout: IG1: 3 IG2: 2 IG3: 0 CG: 2 Adverse effects: Not mentioned.	Between-group comparisons: Muscle strength: Compared with CG: IG1, IG2, and IG3 showed significant improvements in muscle strength (*p* < 0.01). No differences were found between IG1, IG2 and IG3. Body composition: Compared with CG: IG1, IG2, and IG3 showed significant improvements in lean mass. No change in fat mass. Physical Function: Compared with CG: Chair-rise time decreased significantly in IG1, IG2, and IG3 (*p* < 0.01). The highest percentage of decreased was observed in IG3 (30.2 ± 11%). There were significant improvements in the time spent in 6-meter backward walk for the IG groups (*p* = 0.01) Within-group comparisons: Muscle strength: No significant differences were observed (*p* = 0.87) No other measures were reported.
Nemoto et al. (21) 2007 Matsumoto, Japan	246 older adults (63 ± 6 years) (186 women; 60 men) IG1 (*n* = 84; men = 25, 67 ± 4 years; women = 59, 64 ± 6 years) IG2 (*n* = 75; men = 16, 67 ± 5 years; women = 59, 62 ± 5 years) CG (*n* = 87; men = 19, 66 ± 5 years; women = 68, 62 ± 6 years) Design: Randomized controlled trial.	Primary Outcome: Muscle strength: Knee extension and flexion forces (dynamometer) Body Composition: Body weight and BMI	IG1: High-intensity interval walking training. 5 sets (3 min low-intensity walking at 40% VO_2_ peak followed by 3-min high-intensity walking (>70% VO2 peak). Four or more days per week for 5 months. IG2: Moderate intensity continuous walking training: walk (50% VO2peak) 8,000 steps per day. 4 or more days per week for 5 months CG: No walking training	Measurements: At baseline At 5 months Dropout: IG1: 42 IG2: 24 CG: 41 Adverse effects: Not mentioned.	Between-group comparisons: Compared with CG, IG1 showed significant improvements in knee extension and flexion forces (*p* < 0.001) and in body weight (*p* < 0.001) and BMI (*p* < 0.004) in women. Compared with CG, IG2 showed a significant decrease in body weight and BMI (*p* < 0.001) in women. Compared with IG2, IG1 showed significant differences in knee flexion forces in men (*p* = 0.003), women (*p* = 0.02) and total number of participants (*p* = 0.004) Within-group comparisons: IG1 showed significant differences in isometric knee flexion (*p* < 0.001) and extension in women, men and total number of participants (*p* < 0.001), in BMI (*p* = 0.01) and body weight (*p* = 0.02) IG2 showed significant differences from pre-training values in isometric knee flexion in total number or participants (*p* < 0.001) in BMI (*p* < 0.001) and body weight (*p* < 0.001)
Villanueva et al. (22) 2014 California United States	22 men (68 ± 4.1 years) IG (*n* = 11, 65.6 ± 3.4 years) CG (*n* = 11, 70.3 ± 4.9 years) Design: Randomized controlled trial.	Primary Outcome Body composition: (DEXA) Muscle strength: chest press and bilateral leg press exercises with (1-RM) Secondary Outcome Physical Function: Muscle performance (Margaria power test), balance (SEBT) and gait speed (400-m walk)	12 weeks (4 weeks preparatory training + 8 weeks strength training). 36 Sessions (45–60 min), 3 days per week. IG: High intensity strength resistance training with short rest interval (60 s) CG: High intensity strength resistance training with extended rest interval (4 min)	Measurements: Prior to a 4-week control period. At baseline At 4 weeks At 8 weeks At 12 weeks Dropout: IG: 0 CG: 0 Adverse effects: No injuries, illness or personal choice were observed.	Between-group comparisons: Compared with CG, IG showed significant increases in lean body mass (*p* = 0.001), dynamic muscle strength (*p* < 0.001), and muscle performance (*p* < 0.001) Within-group comparisons: After intervention, IG showed significant improvements in lean body mass correlated with muscle strength: chest press (*r* = 0.88, *p* < 0.01), pulldown strength (*r* = 0.68, *p* < 0.05), and single-leg knee extension strength (*r* = 0.69, *p* < 0.05).
Sculthorpe et al. (23) 2017 Scotland United Kingdom	33 sedentary men 56–65 years) IG (*n* = 22, 62.3 ± 4.1 years) CG (*n* = 11, 61.6 ± 5 years) Design: Randomized controlled trial.	Primary outcome: Muscle strength: Peak muscle power (Cycle ergometer) Body composition: TBM, FFM, FM (BIA) Secondary Outcome: Physical function: Static balance (Footscan portable foot pressure plate and stability)	IG: Conditioning exercise: 6 weeks. ≥5 days per week. Sessions of ≥ 30 min. HIIT intervention: 6 weeks. One session every 5 days. 5 min warm-up; 6 x 30 s sprints with 3-min intervals of active recovery. CG: Inactive	Measurements: At baseline At 6 weeks At 6 weeks Dropout: IG: 0 CG:0 Adverse effects: No adverse effects were reported.	Between-group comparisons: Compared with CG, IG showed significant differences in peak muscle power (*p* < 0.01) and lean body mass (*p* < 0.01). Regarding static balance, no significant differences were observed. Within-group comparisons: After the intervention, IG showed significant improvements in peak muscle power (*p* < 0.01), lean body mass (*p* < 0.05), and a significant decrease in total body fat (*p* < 0.05)
Moro et al. (24) 2017 Padua Italy	35 older adults (15 women; 20 men) IG1 (*n* = 18, 64.1 ± 2.3 years; women = 8; men = 10) CG (n = 17, 61.7 ± 4.2 years; women = 7; men = 10) Design: Randomized controlled clinical trial.	Primary Outcome: Body composition: Height and body weight (digital electronic scale), FFM and FM (BIA) Muscle strength: 3–6 RM strength (leg extension, chest press, lat pull down and arm curl)	IG1: 2 months. 2 times per week (45 min). HIIRT (high intensity interval resistance training): 2 series of 6RM at 80% 1RM followed by 20 ″of rest, repetitions to failure, another 20″ of rest, and repetitions to failure. CG: 2 months. 2 times per week TRT (traditional resistance training): 3 series of 8 repetitions at 75% 1RM.	Measurements: At baseline After the intervention Dropout: IG1: 4 CG:8 Adverse effects: Not mentioned.	Between-group comparisons: Strength increased in IG1 and in CG (*p* < 0.001), without significant difference between them. Within-group comparisons: IG1 and CG showed a small Cohen's effect size for body weight: CG (0.01) IG1 (0.02), FFM: CG (0.26) IG1 (0.15), and FM: CG (0.07) and IG (0.11).
García-Pinillos et al. (16) 2017 Jaén, Spain	90 older adults (72 ± 5 years) (64 women; 26 men) IG1 [*n* = 47, 73.50 ± 5.58 years; women = 34 (72.3%), men = 13 (27.7%)] CG [*n* = 43, 72.09 ± 5.78 years; women= 30 (69.8%), men = 13 (30.2%)] Design: Randomized controlled clinical trial.	Primary Outcome: Body composition: BMI, body mass, fat mass, SMM (eight-polar tactile electrode BIA) Muscle strength: Lower-body muscle strength (30-s CST) and upper-body muscle strength Hand-grip strength (hand dynamometer) Secondary Outcome Physical function: Gait speed (GS) and Balance (FreeMed© BASE model baropodometric platform)	IG1: 12 weeks; 3 times per week (35–40 min). HIIT: High-intensity strength training combined with high-intensity interval endurance training Warm-up (5–7 min) High-intensity strength training + high-intensity interval endurance training + High-intensity strength training and cool down (4–5 min) CG: Walking (150–200 min per week at low-moderate intensity)	Measurements: At baseline At 12 weeks Dropout: IG1: 0 CG:4 Adverse effects: No adverse events were reported.	Between-group comparisons: Compared with CG, IG1 showed significant improvements in BMI, fat mass, and SMM (*p* < 0.005). Also showed significant differences in 30-s CST (*p* < 0.001) and hand-grip strength (*p* = 0.048), GS (*p* = 0.007), and length balance (*p* = 0.003). Within-group comparisons: IG1 showed significant interactions in body mass, fat mass, muscle mass, BMI, 30-s CST, hand-grip strength, GS (*p* < 0.001), and balance for ellipse area (*p* = 0.031) and length (*p* < 0.001) CG: No significant differences were observed (*p* ≥ 0.05)
Jiménez-García et al. (25) 2019 Málaga, Spain	82 older adults (68.23 ± 2.97 years) (women 75.61%) IG1 (*n* = 28, 68.23 ± 2.97 years; women 92.3%) IG2 (*n* = 27, 68.75 ± 5.98 years, women 70.8%) CG (*n* = 27, 68.52 ± 6.33 years; women 65.2%) Design: Randomized controlled clinical trial.	Primary Outcome: Muscle strength: Hand-grip strength (hand-grip dynamometer) Body composition: SMM and PBF (BIA) Secondary Outcome Physical Function: Gait speed (TUG)	IG1: High-intensity interval exercise (HIIT). 12 weeks; 2 times per week. Warm-up (10 min); 4 sets of squat activity with suspension 90–95% max HR followed by active rest intervals (90–95% max HR) followed by 3-min active rest intervals (50–70%) and a cool-down (10 min). IG2: Continuous-intensity-training (MIIT). 12 weeks; 2 times per week. Warm-up (10 min); 4 sets of squat activity with suspension 70–50% max HR followed by active rest intervals (70–50% max HR) followed by 3-min active rest intervals (50–70%) and a cool-down (10 min). CG: their daily lifestyle and a guideline to encourage physical activity	Measurements: At baseline At 12 weeks Dropout: IG1: 2 IG2:3 CG:4 Adverse effects: Injuries and other effects were observed.	Between-group comparisons: Compared with CG, IG1 showed significant differences in BMI (*p* < 0.001) and gait speed (*p* < 0.001). Compared with IG2, IG1 showed significant improvements in BMI (*p* = 0.002) and gait speed (*p* < 0.001). Compared with CG, IG2 showed significant differences in BMI (*p* = 0.01). No significant differences were observed for SMM, PBF, or hand-grip strength. Within-group comparisons: IG1 showed significant improvements in hand-grip strength (p = 0.002), gait speed (p = 0.002)
Ballesta-García et al. (26) 2019 Murcia, Spain	54 women (67.8 ± 6.2 years) IG1 (*n* = 18, 66.3 ± 5.44 years) IG2 (*n* = 18, 70 ± 8.76 years) CG (*n* = 18, 67.4 ± 5.71 years) Design: Randomized controlled trial.	Primary Outcome: Body composition: BMI (electronic balance and a height rod) Muscle strength: Hand-grip strength (dynamometer); Upper-limb strength (ACT-30) and lower-limb strength (STS-30) Secondary Outcome: Physical function: Gait speed (TUG) and balance (OLS)	IG1: 18 weeks; 2 times per week (1 h). High-intensity interval training in a circuit program: (Warm-up, HIIT (14–18 point of RPE) and cool-down) IG2: 18 weeks; 2 times per week (1h). Moderate-intensity continuous training: (Warm-up, (9–14 point of RPE) and cool-down) CG: Their physical activity habits.	Measurements: At baseline At 18 weeks Dropout: IG1: 1 IG2:6 CG:6 Adverse effects: 5 subjects presented adverse effects during the study.	Between-group comparisons: Compared with CG, IG1 showed significant improvements in ACT-30 (*p* < 0.001), STS-30 (*p* < 0.001), TUG (*p* < 0.001) and BMI (*p* < 0.001). Compared with CG, IG2 showed significant improvements STS-30 (*p* < 0.001) and TUG (*p* < 0.001). Compared with IG2, IG1 showed significant improvements in 30-second ACT (*p* < 0.001). Within-group comparisons: IG1 showed significant improvements in STS-30 (*p* < 0.001), TUG (*p* < 0.001), ACT-30 (*p* = 0.022), right OLS (*p* = 0.024), and BMI (*p* = 0.035). IG2 showed significant improvements (*p* < 0.001) in STS-30 and TUG. CG showed significant improvements in ACT-30 (*p* < 0.001), STS-30 (*p* < 0.001), TUG (*p* = 0.016), and BMI (*p* = 0.019).

ACT-30, 30-second Arm Curl Test; BIA, Bioelectrical Impedance Analysis; BMI, Body Mass Index (weight in kilograms divided by the square of height in meters); CG, Control Group; CSA, cross-sectional area; 30-CST, 30 second chair stand test; DXA, Dual energy X-ray absorptiometry; FFM, Fat-Free Mass; FM, Fat Mass; GS, Gait speed test; HR, Heart rate; IG, Intervention Group; OLS, one-leg standing test; RPE, rating of perceived exertion; PBF, body fat; SEBT, Star Excursion Balance Test; STS-30, 30-second sit-to-stand; SMM, Skeletal muscle mass percentage; TBM, Total body mass; TUG, Timed Up-and-Go Test; UGS, Usual gait speed; VO2 peak, peak oxygen consumpt.

Two articles enrolled only men (22, 23), one article enrolled only women (26), and five articles included both genders. A total of 615 participants took part in the eight articles included in this systematic review, and out of those 65.04% were women. Table 2 shows mean age by groups. The types of exercises reported were high-intensity interval aerobic or resistance training, either alone or combined. Out of the eight RCTs included, only four studies used active rest in their interventions (21, 23, 25, 26). The duration of the interventions was measured in weeks in 6 of the 8 studies, with an average of 14 (range: 6–24), including several weekly sessions, with a distribution of 12 weeks in the articles by Villanueva et al. (22), Jiménez-García et al. (25) and García-Pinillos et al. (16), 24 weeks in the article by Taaffe et al. (20), 18 weeks in Ballesta-García et al. (26), and 6 weeks in Sculthorpe et al. (23). Meanwhile, the articles by Moro et al. (24) and Nemoto et al. (21) reached lengths of 2 and 5 months, respectively. The dropout rate was 24.7% (152/615 participants). Two of the RCTs (25, 26) reported adverse effects, three other articles (16, 22, 23) did not register adverse effects, and the articles by Taaffe et al. (20), Nemoto et al. (21) and Moro et al. (24) did not provide any statement regarding adverse effects.

### Outcomes

#### Body composition

Body composition was assessed by whole-body dual-energy X-ray absorptiometry in two of the articles (20, 22), Bioelectrical Impedance Analysis was used in four articles (16, 23–25), which also employed an electronic scale and a height rod. Four studies reported that high-intensity strength-resistance training improved lean body mass measurements (16, 22, 23, 26). In two of those BMI also improved (16, 26), and two others showed a significant decrease in total body fat after the intervention (16, 23). Two articles looked into body weight differences within a high-intensity interval resistance group and reported improvement in this outcome. Moro et al. (24) also found improvements in fat mass and lean body mass, while Nemoto et al. (21) reported a decrease in body weight.

When intervention groups were compared with controls, changes in body composition were observed. Three studies that included high-intensity strength-resistance training reported improvements in lean body mass compared with a control group (16, 22, 23). Taaffe et al. (20) also found significant improvements in lean mass in all intervention groups compared with controls, for which this outcome did not change. Two other articles (21, 26) reported significant differences in BMI, and one of them observed improvements in body weight (21). Finally, Jiménez-García et al. (25) found that a group engaged in a training program including high-intensity intervals of TRX suspension exercises improved their outcomes more effectively than a continuous-intensity interval training group or a control.

#### Muscle strength

Muscle strength was assessed using hand-grip, knee extension, knee flexion, lower-body and upper-limb strength as proxies. Measurements were performed with the help of a dynamometer, a cycle ergometer, repetition maximums, the 30-s Arm Curl Test, and the 30-s Chair Stand Test.

Regarding within-group comparisons, six articles found improvements in muscle strength after high-intensity interval resistance training (16, 21–23, 25, 26). As far as between-groups comparisons were concerned, results were mixed. Jiménez-García et al. (25) did not observe increases in muscle strength after 12 weeks of high-intensity interval suspension training compared with moderate-intensity training and a control group. However, four studies reported significant increases in muscle strength after high-intensity interval strength-resistance training compared with a control group (16, 22, 23, 26). Out of these four studies, one reported significant differences in upper-limb strength compared with moderate-intensity continuous training (26). Furthermore, two articles showed an increase in muscle strength after a high-intensity interval resistance training intervention, compared with an inactive control group (20, 21). Taaffe et al. (20) failed to find differences between performing the intervention one, two, or 3 days per week, and Nemoto et al. (21) found significant improvements in muscle strength compared with moderate-intensity training. On the other hand, Moro et al. (24) reported significant increases in muscle strength in a high-intensity interval resistance training and in a traditional resistance training, but no differences were apparent between these two forms of exercise.

#### Physical function

Physical function included the domains of balance, gait speed, and muscle performance. Balance was assessed through the Star Excursion Balance Test, the 6-meter backward tandem walk, the Footscan portable foot pressure plate and stability software, ratings of perceived exertion, and the FreeMed© BASE model baropodometric platform. Gait speed was evaluated using the 400-meter walk test, the Timed Up-and-Go Test, and the gait speed test. Finally, muscle performance was assessed through the Margaria power test and the chair-rise test.

Out of the eight articles included in this systematic review, six assessed physical function outcomes. Jiménez-García et al. (25), García-Pinillos et al. (16), and Ballesta-García et al. (26) reported significant within-group differences in gait speed, also in comparison with a moderate-intensity interval training group. Jiménez-García et al. (25) and Ballesta-García et al. (26) also observed significant differences compared with a control group that stuck to their usual physical activity habits. Five of the articles assessed balance (16, 20, 22, 23, 26). García-Pinillos et al. (16) reported improvements in ellipse area balance and length balance in within-group comparisons, and also in length balance compared with a control group. Ballesta-García et al. (26) also reported significant differences in balance in within-group comparisons. However, three other articles did not observe significant differences in within-group comparisons (20, 22, 23), but one of them reported significant differences in balance, assessed by the 6-meter backward tandem walk, compared with a control group (20). Two articles measured muscle performance, and both reported improvements compared with control groups after their high-intensity interval resistance training (20, 22).

## Discussion

The goal of this systematic review of control trials was to analyze the effects of high-intensity interval training on the body composition, muscle strength, and physical function of healthy elderly individuals.

Aging is associated with increased odds of developing one or several financially costly conditions (27). On the other hand, the consequences of a sedentary lifestyle have become a public health problem across all age groups. Physical inactivity during the aging process accelerates the loss of muscle strength and function, increases fat mass, decreases quality of life, and increases the risk of mortality (28). All the reasons stated above highlight the need to promote and achieve among the population an active attitude toward aging, which is why recent years have witnessed a sharp increase in the number of studies looking into the effects of a variety of programs centered on physical activity.

A total of eight articles were included in this systematic review. Regarding within-group results all of them, with the exception of the one carried out by Taaffe et al. (20), reported improvements in some of the parameters analyzed. Improvements in body composition were observed in six of them (16, 21–24, 26), and increases in muscle strength were observed in seven of the eight (16, 21–26). Regarding physical function, only six of the eight articles considered this parameter, with two reporting improvements in gait speed (25, 26) and one finding improvements in balance (16).

As for the frequency of training sessions, only Taaffe et al. (20) evaluated the weekly frequency with which HIIT was performed. Regarding body composition and muscle strength, they did not find differences between training 1, 2, or 3 days per week. Other investigations are in line with these results, having concluded that in the first phases of training the volume or frequency of the same does not significantly affect strength adaptations (29, 30). Despite this, when subjects find themselves at a more advanced phase of training, frequency becomes a relevant factor in the achievement of increased muscle strength (29). Regarding the secondary outcomes, it has been shown that engaging in HIIT 3 days per week is more effective than 2-day or 1-day-per-week regimes regarding muscle performance, whereas no differences were found in balance outcomes.

The results of this systematic review suggest that HIIT-based interventions have beneficial effects on body composition. However, when a comparison was made between HIIT and moderate-intensity training groups, contradictory results were observed. On the one hand, two articles found significant improvements in BMI (16, 25), and one of them also found significant improvements in fat mass (16). On the other hand, two other articles did not find any significant difference (21, 24). These results are in line with those obtained by Wewege et al. (17) in their systematic review and meta-analysis involving overweight and obese adults aged 18–45 years, in which it was concluded that both interventions present similar results across all body composition measures. It was proposed that HIIT might be a better option, as it is more time-effective in weight management programs. Furthermore, in this regard Moro et al. (24) explained that the intensity achieved during HIIT may not have been enough to induce significant fat loss, but succeeded at preventing fat gain more effectively than traditional resistance training.

Aging leads to a decrease in the elements necessary for axonal regeneration (31). This is likely to influence corticocortical and corticospinal connectivity and cause a loss of muscle strength (32). As a matter of fact, this deterioration of maximum muscle force and of its rate of development has also been observed in professional athletes (33). These results are in line with those reported in the studies included in this review, in which HIIT was shown to improve muscle strength in untrained subjects when compared with people who did not perform any physical activity. Be that as it may, two authors (24, 25), while failing to find statistically significant differences, did register some improvement within the HIIT group, which indicates that if muscle strength did not increase at least it was not decreased.

On the other hand, Onambélé-Pearson et al. (34) determined, in their study on the influence of exercise intensity among older people that as far as muscle strength is concerned high-intensity training turns out to be more effective than comparable low-intensity regimes. Their observations agree with the conclusions obtained by two (21, 26) of the three (21, 25, 26) articles included in this review in which HIIT was compared with MIIT. However, although Jiménez-García et al. (25) did not agree with these results, they did report an increase in the muscle strength of participants who engaged in HIIT. Such differences between groups of high-intensity and moderate-intensity training may have been due to the duration of the interventions, which in the case of Jiménez-García et al. (25) was of 12 weeks in contrast to those carried out by Nemoto et al. (21) and Ballesta et al. (26), which took 5 months and 18 weeks respectively. Another potential reason behind such differences may lie in the measurement tools employed: in the intervention devised by Jiménez-García et al. (25) hand-grip strength was evaluated using a dynamometer, similarly to Ballesta-García et al. (26), in which no differences were found in muscle strength. However, differences appeared when upper-limb strength was assessed by means of the 30-s push-up test. These conclusions are particularly relevant given that greater muscle strength in the upper limbs is associated with improved quality of life in women over 60 years of age (35). In addition, current scientific evidence also provides evidence concerning the association of grip strength with the function of the upper extremities, bone mineral density, fractures, falls, and increased risk of mortality among older people. This appears, therefore, to be a particularly relevant parameter which should be analyzed more thoroughly in future research (36).

In the analysis of physical function, significant improvements were observed in muscle performance (20), gait speed (25), and balance (26) with respect to their control group. These last results are in contradiction with those of Sculthorpe et al. (23), which did not find significant improvements in balance. This could be attributed to the type of intervention, as it was performed on a cycle ergometer with five stability points which may have hampered the recording of balance improvements. It has been reported that improvements to physical function may reduce the risk of falls (37). In addition, three (16, 25, 26) articles looked at the physical function of HIIT groups in comparison with moderate-intensity interval training, and their results also turned to be contradictory in that regard. On the one hand, Jiménez-García et al. (25) reported differences in gait speed, and García-Pinillos et al. (16) in length balance, in contrast to the results of the study by Ballesta-García et al. (26), in which no significant differences were found for either balance or gait speed.

There are some limitations to this systematic review that should be noted. The heterogeneity of the measurement instruments for the variables under analysis in each of the articles renders a meta-analysis impossible. Another limitation concerns the fact that the effects of the interventions were only measured in the short term. In addition, the differences between types of HIIT, the length of the series, the number of repetitions, and the duration of the sessions may be considered as limitations, since such factors may influence the results. Future HIIT interventions should consider longer intervention periods, as well as looking into the long-term effects of their interventions in order to better understand the beneficial effects of this type of training on general health, and particularly on muscle strength, body composition, and physical function.

## Conclusion

After conducting a systematic review of published data to assess the effects of HIIT on the body composition and muscle strength of middle-aged and older adults, HIIT was found to be an effective tool for improving body composition and increasing muscle strength; however, regarding physical function, the results do not allow for clear conclusions and although it seems that HIIT may have positive effects on this parameter, its disparity indicates that caution should be used when drawing a firm conclusion. On the other hand, when HIIT is compared with other types of training, such as continuous training of moderate intensity, it is not clear if HIIT is more effective, due to the limited published evidence in this regard, the great variety in the methodology used in the studies and the ambiguity of the data provided make it impossible to draw a firm conclusion; nevertheless it appears that both types of training have beneficial effects on body composition, muscle strength and physical function in a population of middle-aged and older people. It is important to emphasize that more quality randomized controlled trials with an adequate sample size are still needed to lead to a correct understanding of the effects of HIIT on the variables studied in the short and long term in among middle-aged and older adults. Likewise, more studies are required to determine if HIIT is a better, worse or equivalent alternative to other types of physical training to confirm these results. This step is essential for advising specific training characteristics that will improve body composition and maximize physical function and muscle strength.

## Data availability statement

The original contributions presented in the study are included in the article/supplementary material, further inquiries can be directed to the corresponding author/s.

## Author contributions

Conceptualization: YC-C and MA-T. Methodology: AA-A and FH-C. Performing literature review and synthesis of literature: MC-F, AA-A, and RF-C. Quality assessment: JJ-G, FH-C, and MA-T. Writing—original draft preparation: MC-F, AA-A, and MA-T. Writing—reviewing and editing: AM-A, YC-C, JJ-G, and RF-C. Funding acquisition: FH-C and AM-A. All authors have read and agreed to the published version of the manuscript.

## Funding

This work was partly supported by project 1260735 from the 2014 to 2020 Operational Programme FEDER in Andalusia.

## Conflict of interest

The authors declare that the research was conducted in the absence of any commercial or financial relationships that could be construed as a potential conflict of interest.

## Publisher's note

All claims expressed in this article are solely those of the authors and do not necessarily represent those of their affiliated organizations, or those of the publisher, the editors and the reviewers. Any product that may be evaluated in this article, or claim that may be made by its manufacturer, is not guaranteed or endorsed by the publisher.
